# DNA methylation‐wide association study of prevalent and incident dementia in the US Health and Retirement Study

**DOI:** 10.1002/alz70855_103847

**Published:** 2025-12-24

**Authors:** John Dou, Scarlet M Cockell, Herong Wang, Lindsay H Ryan, Matthew Zawistowski, Erin B Ware, Kelly M. Bakulski

**Affiliations:** ^1^ University of Michigan, Ann Arbor, MI, USA; ^2^ University of Michigan/ Insitute for Social Research, Ann Arbor, MI, USA; ^3^ Michigan Alzheimer's Disease Research Center, Ann Arbor, MI, USA

## Abstract

**Background:**

Dementia is a progressive neurodegenerative disease and studies of peripheral blood DNA methylation biomarkers, which may have utility for early risk detection or monitoring progression, have been limited by sample size. We analyzed cognitive impairment and DNA methylation in the United States Health and Retirement Study (HRS), a large longitudinal cohort study nationally representative of US adults over the age of 50.

**Method:**

Site specific DNA methylation was measured in blood collected in the 2016 HRS wave. We performed cross‐sectional analysis using 2016 HRS methylation and cognitive data, comparing participants with cognitive impairment but not dementia (CIND) and with dementia to those with normal cognition, adjusting for age, sex, education, cell proportions, genetic principal components, and APOE‐ε4 carrier status. Second, we performed prospective analysis restricted to persons with normal cognition at baseline and examined association between baseline DNA methylation and any cognitive impairment with up to four years of follow‐up.

**Result:**

In cross‐sectional analysis (*n* = 3,395), most methylation sites nominally associated (*p* <0.01) with CIND (sites=5,962) or dementia status (sites=10,115) had higher DNA methylation compared to those with normal cognition (80.4% and 81.6%, respectively). CIND associated sites were enriched (adjusted‐p<0.05) for pathways related to cell connectivity and ion transport. Dementia associated sites were enriched in pathways related to cell movement, neuron cell structure, and transcription. In prospective analysis (*n* = 2,424 cognitively normal at baseline), among sites nominally associated with development of any cognitive impairment (sites=7,228), 85.2% had higher DNA methylation compared to those retaining normal cognition. These sites were enriched in pathways related to cell connectivity. Between the cross‐sectional CIND and dementia, and prospective any impairment analyses, 22 CpGs were nominally associated in all analyses. The site cg25144382, which is not annotated to a gene, had 1.3% higher methylation in prevalent CIND (*p* = 5.1x10‐5), 2.7% higher methylation in prevalent dementia (*p* = 6.0x10‐5), and 1.8% higher methylation in incident impairment (*p* = 1.3x10‐5).

**Conclusion:**

Blood DNA methylation may be a potential biomarker for incident cognitive status in older adults.

